# Assessment of brainstem function and haemodynamics by MRI: challenges and clinical prospects

**DOI:** 10.1259/bjr.20220940

**Published:** 2023-09-18

**Authors:** Owen Bleddyn Woodward, Ian Driver, Stefan Theodor Schwarz, Emma Hart, Richard Wise

**Affiliations:** 1 Cardiff University Brain Research Imaging Centre (CUBRIC), Cardiff University, Cardiff, United Kingdom; 2 University Hospital of Wales, Cardiff, United Kingdom; 3 University of Bristol, Bristol, United Kingdom; 4 Institute for Advanced Biomedical Technologies, University "G. d'Annunzio" of Chieti-Pescara, Chieti, Italy; 5 Department of Neurosciences, Imaging and Clinical Sciences, University "G. d'Annunzio" of Chieti-Pescara, Chieti, Italy

## Abstract

MRI offers techniques for non-invasively measuring a range of aspects of brain tissue function. Blood oxygenation level dependent (BOLD) functional magnetic resonance imaging (fMRI) is widely used to assess neural activity, based on the brain’s haemodynamic response, while arterial spin labelling (ASL) MRI is a non-invasive method of quantitatively mapping cerebral perfusion. Both techniques can be applied to measure cerebrovascular reactivity (CVR), an important marker of the health of the cerebrovascular system. BOLD, ASL and CVR have been applied to study a variety of disease processes and are already used in certain clinical circumstances. The brainstem is a critical component of the central nervous system and is implicated in a variety of disease processes. However, its function is difficult to study using MRI because of its small size and susceptibility to physiological noise. In this article, we review the physical and biological underpinnings of BOLD and ASL and their application to measure CVR, discuss the challenges associated with applying them to the brainstem and the opportunities for brainstem MRI in the research and clinical settings. With further optimisation, functional MRI techniques could feasibly be used to assess brainstem haemodynamics and neural activity in the clinical setting.

## Introduction

The brainstem serves as a conduit for ascending and descending nerve tracts and it contains cranial nerve nuclei as well as autonomic centres that control blood pressure, heart rate and respiration. It is a critical element of the central nervous system, but it is more challenging to study using magnetic resonance imaging (MRI) than many other regions of the brain. While routine clinical MRI usually aims to identify structural abnormalities, MRI is also capable of non-invasively interrogating the function of the central nervous system. Blood oxygenation level dependent (BOLD) functional MRI (fMRI) is a method of assessing neuronal function, and arterial spin labelling (ASL) is a non-invasive method of assessing cerebral perfusion without the administration of an exogenous contrast agent. Both ASL and BOLD can also be used to measure cerebrovascular reactivity (CVR), an important marker of the health of the cerebrovascular system.

The application of these techniques to evaluate the brainstem is largely confined to research at the moment, but brainstem networks involved in pain processing,^1,2^ arousal^3,4^ and autonomic activity^5–8^ have been mapped to various extents. The increasing availability of 3 Tesla scanners may open the door to the future clinical application of brainstem fMRI. This review is aimed at clinicians who are interested in applying functional MRI methods in their clinical or research practice to examine patterns of changing neuronal activity in the brainstem and assess haemodynamic function. We introduce the technical details underpinning the techniques and their availability, discuss the challenges associated with applying them to the brainstem and describe the future potential for applying brainstem functional MRI in the clinical setting.

## Functional neuroanatomy of the brainstem

**Figure 1.**
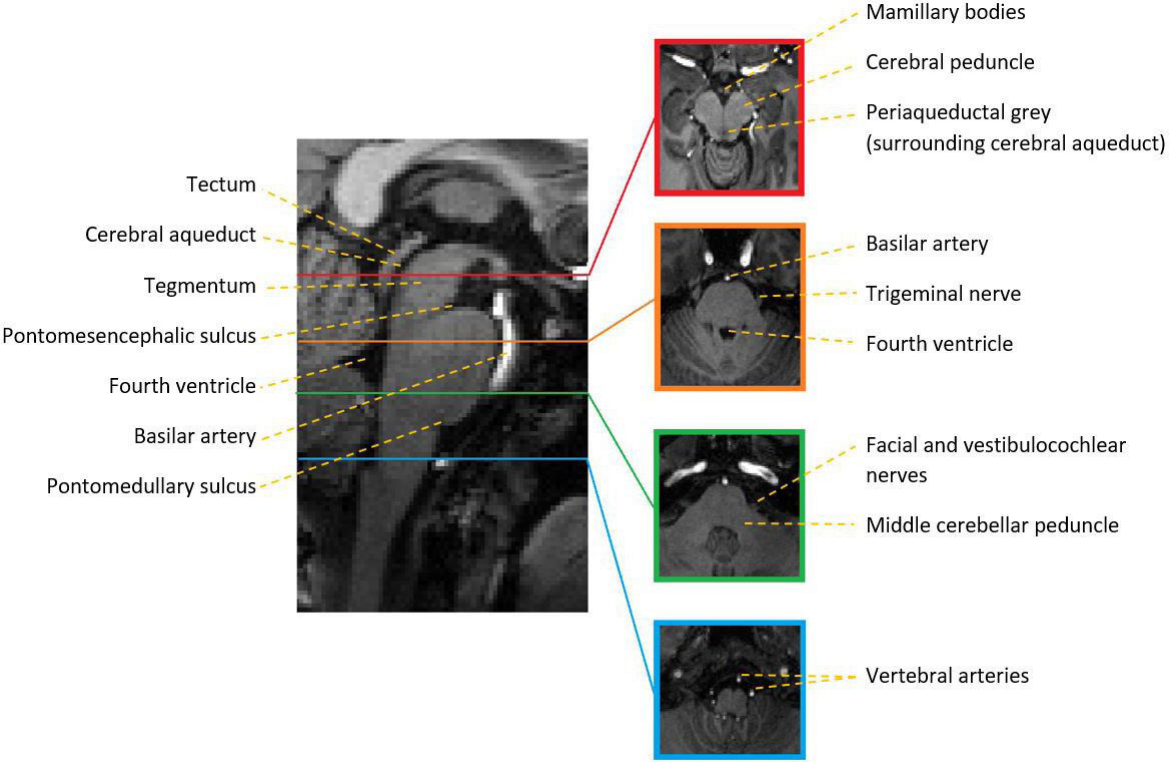
Brainstem anatomy outlined on *T_1_*-weighted mid-sagittal and selected axial cross-sections through the brainstem.

The brainstem is a small structure^9^ consisting of three regions, the midbrain, pons and medulla (Figure 1). The average diameter of its smallest subdivision, the medulla, is 14 mm^10^ and many brainstem nuclei measure less than 1 mm in diameter^11,12^ The cerebral aqueduct runs through the midbrain, connecting the third and fourth ventricles and separating the midbrain into the tectum dorsally, and the tegmentum and cerebral peduncles, which connect the midbrain to the cerebral hemispheres, ventrally. Notable structures within the midbrain include the substantia nigra (involved in reward and movement), the red nucleus (also involved in motor coordination), and the periaqueductal grey (involved in pain processing). Cranial nerves III and IV arise from the midbrain surface. The inferior boundary of the midbrain is defined by the pontomesencephalic sulcus. Below this lies the pons, the largest subdivision. The ventral pons contains motor nuclei which communicate with the cerebellum via the middle cerebellar peduncle. Dorsally, the phylogenetically older tegmentum houses parts of the reticular formation (involved in arousal and wakefulness). The trigeminal nerve (cranial nerve V) arises from the pons. Cranial nerves VI, VII and VIII arise from the pontomedullary sulcus, which defines the boundary between the pons and medulla. This is the most caudal subdivision of the brainstem, and it merges with the spinal cord inferiorly. The pyramids, which contain motor fibres from the prefrontal cortex, decussate in the ventral medulla, and cranial nerves IX, X, XI and XII emerge from its surface. While the precise anatomy of the brainstem vasculature is somewhat variable,^13^ most of the arterial supply to the brainstem is from the vertebrobasilar arteries.

## Arterial spin labelling

### Physical principles

**Figure 2.**
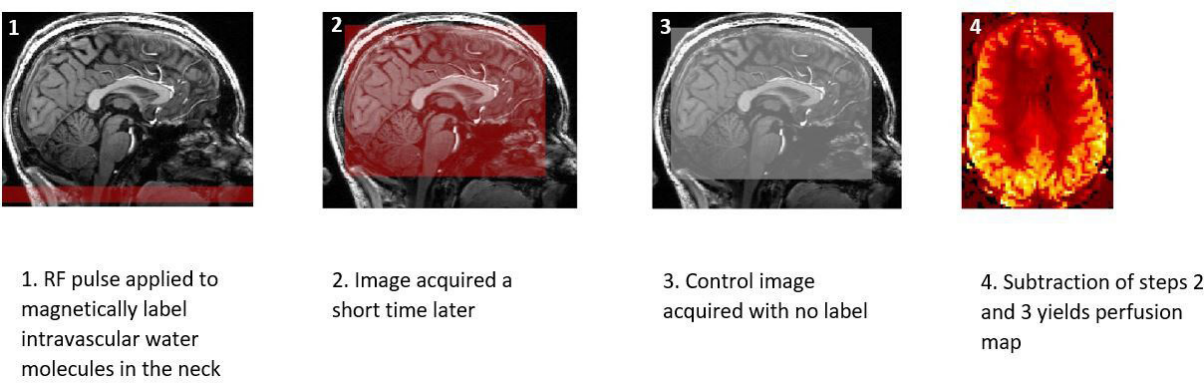
Simplified representation of step-by-step process of generating a cerebral perfusion map using arterial spin labelling.

ASL involves magnetically 'labelling' the water in a bolus of intravascular blood, which then travels into the brain parenchyma enabling an estimation of perfusion to be made through the application of a kinetic model.^14^ A radiofrequency inversion pulse is applied to invert the magnetisation of blood in the carotid and vertebral arteries, then after a short time delay (termed the post-label delay (PLD)) to allow it to travel to the brain, a ‘tag’ image is acquired. A 'control' image is then acquired, identical to the ‘tag’ image in all respects apart from the inversion effect of the labelling pulse. Subtraction of the 'tag' and 'control' images ideally leaves only signal from inverted blood, which represents perfusion information (Figure 2).

There are two main labelling techniques which are currently in use. Pseudocontinuous ASL (PCASL) involves the application of a train of radiofrequency (RF) pulses together with a magnetic field gradient (applied in the direction of blood flow). As blood flows along the field gradient, the precession frequency of hydrogen nuclei sweeps through resonance with the applied RF pulse. The alternate approach, pulsed ASL, involves the application of a single RF pulse to the labelling volume which almost instantaneously inverts the net magnetisation within that volume. The details of these techniques can be found in previous publications.^15,16^

**Figure 3.**
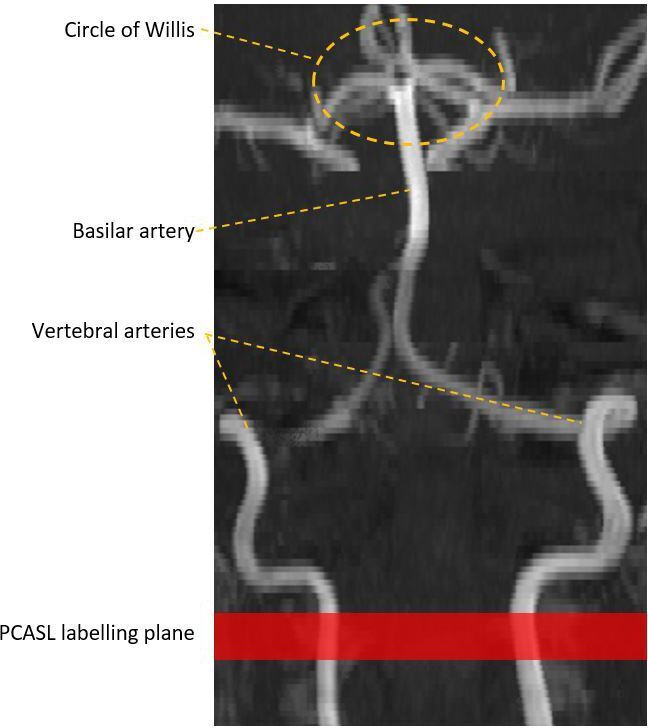
Time-of-flight MRI angiogram of the vertebrobasilar arteries demonstrating representative example of label location for brainstem ASL.

In order to achieve efficient inversion of blood as it flows through the labelling plane, it is important to apply the labelling pulse perpendicular to the direction of blood flow in the arterial vessels of the neck. This is especially important for brainstem ASL because of the tortuous course of the vertebral arteries in the superior part of the neck (Figure 3). An angiogram can be acquired to help locate the labelling plane (Figure 3).^15^ An alternative labelling method, velocity-selective ASL (VSASL), involves labelling blood according to its flow velocity rather than its spatial location.^17^ Blood within arterioles supplying the brain parenchyma can therefore be directly labelled. This helps to reduce the signal decay during transit and is less sensitive to the confounding effects of pathology that involves slow or collateral blood flow. The disadvantage of VSASL is that SNR is generally slightly lower than PCASL, and label flow velocity must be accurately targeted.^18^

The magnitude of the difference in signal between the “tag” and “control” images is of the order of 1% of the static tissue water signal.^15^ ASL is, therefore, an inherently low SNR technique. Any form of motion (head movement, physiological tissue displacements or distortions due to noise) between the acquisition of tag and control pairs will have a significant detrimental effect on SNR.^19^ Unfortunately, the brainstem is especially susceptible to such sources of noise, as discussed below. The magnitude of the signal due to such sources of variation is proportional to the signal in the background (unsubtracted) images and is, therefore, potentially much greater than the perfusion signal. Background suppression aims to minimise the signal that originates from static tissues, therefore reducing the effects of any such tissue motion in the image and improving the SNR of the final perfusion image. Background suppression is performed by applying a series of inversion pulses before the acquisition of each image volume. However, the technique is less than 100% efficient and can reduce by as much as 5% the ASL signal.^15^ Post-processing techniques such as motion correction can also be applied, but good patient compliance within the scanner is especially important for obtaining good quality data at the outset.

The time it takes for blood to travel from the labelling plane to brain tissue is known as the arterial transit time (ATT), while the time between labelling and imaging readout is termed the post-labelling delay (PLD). If the PLD is too short, labelled blood might still reside within the basilar artery, which lies in close opposition to the pons, confounding CBF measurements. The PLD needs to be long enough to allow the labelled blood bolus to perfuse into brain tissue. However, the labelled signal decays with T1 relaxation, so longer PLDs reduce the measurement sensitivity and increase acquisition time. Therefore, a PLD that is slightly longer than the longest ATT is recommended^15^ so that most of the labelled blood has reached the cerebral microvasculature before the ‘tag’ image is acquired.

ATT is likely to vary somewhat between individuals, due to variations in flow dynamics and vascular anatomy, or due to vascular pathology.^20^ Ideally, the PLD would be tailored to each individual participant according to their ATT. Multi-PLD ASL involves acquiring ASL data at multiple delay times and enables the kinetic curve of the labelled water to be modelled. This approach allows an ATT to be estimated and used to generate perfusion values which are less sensitive to differences in ATT between brain regions and individuals.^21^ However, multi-PLD ASL SNR is generally lower because fewer tag-control pairs can be acquired per unit time compared to single-PLD acquisitions.^15^

The intrinsic SNR of ASL is higher at 3T than 1.5 T, and T1 relaxation time is longer, enabling a sufficiently high resolution to delineate regional perfusion differences in the brainstem. Brainstem ASL is, therefore, much more technically feasible in the clinical setting with the increasing prevalence of 3T scanners.

### Applications

ASL has not yet achieved widespread application in the clinical setting. However, it offers a potentially useful alternative to techniques that involve ionising radiation such as CT and PET.^15^ In acute stroke, CT perfusion or dynamic susceptibility contrast (DSC) MR^22^ can be used to delineate areas of potentially salvageable brain tissue to select candidates for mechanical thrombectomy. ASL has been shown to be comparable to DSC MR methods.^23–25^ A typical single-PLD ASL acquisition is quick (taking approximately 5 min) and can generate a map of relative cerebral blood flow capable of demonstrating within-subject regional perfusion differences, without the need for complex post-processing methods. ASL could, therefore, serve as part of a standard neuroimaging protocol in assessing patients presenting with symptoms of acute ischaemic stroke. The sensitivity of ASL techniques to pathology that affects the arterial transit time, such as arterial occlusion or stenosis that occurs in ischaemic stroke, can also be exploited to identify collateral supply to hypoperfused regions. Residual labelled blood within the macrovascular circulation will be identified in these areas on perfusion maps as hyperintensities not conforming to the expected distribution of brain perfusion. ASL is potentially more sensitive than time-of-flight MR angiography in detecting arteriovenous malformations and shunts because of the high concentration of labelled blood that flows through these malformations.^26^

ASL is a useful alternative to DSC MRI in assessing the haemodynamics of primary and metastatic brain tumours.^26^ ASL-derived CBV maps can aid in differentiating between low and high-grade tumours, as well as being useful in post-treatment surveillance for high-grade transformation. CBV maps are also useful in differentiating disease progression and response from treatment-related changes (pseudoprogression and pseudoresponse, respectively).^27^ ASL does not require vascular access or intravenous contrast administration, which is especially desirable in the paediatric population where there is uncertainty surrounding the potential long-term effects of gadolinium accumulation in the brain.^23,28^

Cerebral perfusion and metabolism have been shown to be coupled,^29^ and ASL could be an adjunct to PET in identifying reductions in cerebral perfusion or metabolism in early Alzheimer’s disease.^26^ It is also capable of identifying perfusion alterations at epileptogenic foci,^30^ even in the absence of structural abnormalities.

So far ASL has predominantly been applied to study cortical perfusion. However, the feasibility of measuring regional brainstem perfusion at 3T has been demonstrated, and brainstem ASL has been applied in various research settings. These include the demonstration of differential effects of methylphenidate and atomoxetine on regional CBF, including the midbrain,^31^ the study of CBF changes in neonates with hypoxic ischaemic encephalopathy^32,33^ and in differentiating between hereditary ataxia and spastic paraplegia.^34^ With the increasing availability of high-field 3 Tesla MRI in the clinical setting, the applications of ASL described above could all feasibly be applied in the brainstem, and the main vendors are able to supply one or more of the described variants of ASL sequences on the MRI systems that they offer.

## BOLD functional magnetic resonance imaging

### Physical principles

Blood oxygen level dependent (BOLD) fMRI is based on localised temporal variation in MRI signal due to changes in local concentrations of deoxyhaemoglobin in brain tissue. Oxygenated haemoglobin in the arterial circulation is diamagnetic. Conversely, deoxyhaemoglobin, that is found in higher concentration on the venous side-of the circulation, is paramagnetic and induces local static magnetic field inhomogeneities which lead to accelerated MRI signal decay caused by a loss of phase coherence (T2* relaxation). Increased neuronal activity leads to increased cerebral blood flow (CBF) in the local capillary network via a process known as neurovascular coupling.^35^ The fractional increase in CBF exceeds the fractional increase in tissue demand for oxygen (cerebral metabolic rate of oxygen consumption or CMRO_2_), and therefore, somewhat counterintuitively, the oxygen extraction fraction (OEF) from the arterial circulation is lower in regions of higher metabolic activity than in less metabolically active regions. Therefore, in metabolically active regions, the relative concentration of deoxyhaemoglobin is lower, T2* relaxation time is prolonged, and the MRI signal is increased,^14^ normally marking an increase in neuronal activity.

The BOLD signal arises from the haemodynamic response to changes in activity of excitable brain tissue; it is not a direct measure of neuronal activity. Fluctuations in regional neuronal activity can be inferred from the BOLD signal response. Neuronal activity requires energy, and it is currently thought that most of the energy demands of neurons is related to replenishment of postsynaptic neurotransmitters after neuronal firing.^14^ Ultimately, the BOLD signal is more closely correlated with the relatively slowly fluctuating local field potential that represents the electrophysiological changes in groups of neurons, rather than the action potentials of individual neurons.^36^

Changes in CBF, CMRO_2_ and cerebral blood volume (CBV) all contribute to a change in BOLD signal. An increase in CBF tends to increase BOLD signal, whilst an increase in CMRO_2_ or CBV decreases BOLD signal.^14^ Vasoactive stimuli, such as carbon dioxide, induce changes in BOLD signal by modifying CBF, to a first approximation, independently of neuronal stimuli.^37^ BOLD fMRI has been widely used to infer changing neuronal activity in the brain but can also be useful in studying cerebral haemodynamics, as discussed below.

### Applications

Since its inception in the early 1990s,^14^ BOLD fMRI has become the predominant MRI method of interrogating neural activity in the brain. Other non-invasive functional neuroimaging techniques such as electroencephalography (EEG) and magnetoencephalography (MEG) have a higher temporal resolution than BOLD. However, they are predominantly sensitive to cortical activity and have a low spatial resolution. BOLD can be used to probe whole-brain neural activity with a sub-millimetre resolution.

**Figure 4.**
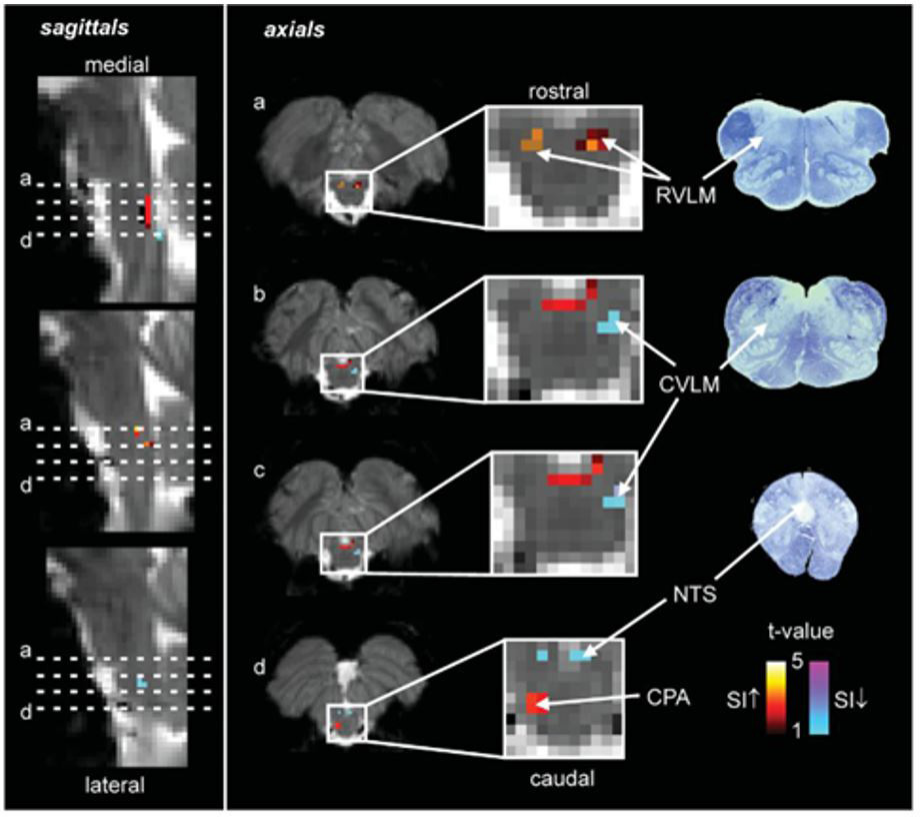
Macefield et al^6^ demonstrated functional activation in several medullary regions involved in autonomic supply to the cardiovascular system including the rostral ventrolateral medulla (RVLM), caudal ventrolateral medulla (CVLM), caudal pressor area (CPA) and nucleus tractus solitarius (NTS). Figure reproduced from ‘Real-time imaging of the medullary circuitry involved in the generation of spontaneous muscle sympathetic nerve activity in awake subjects’^6^ under Creative Commons Attribution 3.0 International License.

The increasing availability of 3 Tesla scanners in clinical practice, and the associated gain in signal-to-noise ratio and spatial resolution, has opened the door to the application of BOLD to study the brainstem.^38^ BOLD has been applied to study regions of the brain involved in pain processing^1,2^ including the midbrain neuronal response to visually triggered migraine.^39^ The subcortical components of the default mode network (a network defined by coherent fluctuations of BOLD signal that appears active during wakeful rest) have been mapped using BOLD fMRI, including parts within the midbrain.^4^ Brainstem neuronal activity in response to deep brain stimulation of the subthalamic nucleus (STN) in patients with Parkinson’s disease has been demonstrated,^40^ including recently in the intra-operative setting.^41^ Several brainstem autonomic networks have been mapped and studied, including those integral to regulation of systemic blood pressure^5–7^ (Figure 4), respiration^8,42^ and swallowing.^43^ Visual processing networks in the brainstem have also been mapped.^44^ BOLD EPI sequences are available for clinical scanners with which these techniques can be implemented. Whilst it is not currently feasible to diagnose network dysfunction in the individual patient, this might develop with higher field strengths (7T and beyond).

In the clinical setting, BOLD fMRI is used for presurgical planning to identify areas of functional activity that might be affected by surgery. Preoperative BOLD fMRI helps to guide the surgical approach and guide the use of intraoperative cortical stimulation in brain tumour surgery.^45^ Brainstem tumours are more common in children than in adults and whilst some are treated surgically, they are often unresectable.^46^ When surgical resection is attempted, diffusion tensor imaging (DTI), a microstructural technique that can be used to map the white matter tracts in the brainstem, has been shown to be useful in pre-operative planning^47,48^ but it is not a reliable predictor of neurological deficits.^48^ The development of brainstem BOLD could serve as an adjunct to DTI in pre-operative planning and potentially enable better predictions of neurological outcomes by accurately localising areas of important functional activity.

One limitation of the application and interpretation of BOLD fMRI in the clinical setting is the potential for decoupling of the neuronal and haemodynamic response. Brain pathology such as vascular malformations, areas of cerebral infarction or mass lesions can disrupt the mechanisms which underpin the BOLD response. This is especially problematic when BOLD fMRI is used for cortical mapping during surgery, where neurovascular uncoupling can lead to areas of false-negative BOLD response, potentially guiding the surgeon to unnecessarily resect areas of eloquent cortex.^49^

The acquisition of brainstem BOLD data necessitates physiological monitoring and correction, as discussed below. Despite these technical demands, brainstem BOLD does have potential as a clinical tool. Accurate functional localisation of brainstem nuclei could help in guiding placement of deep brain stimulators, as well as in guiding brainstem surgery to limit damage to important brainstem nuclei.

## Cerebrovascular reactivity

### Physical principles

CVR represents the capacity of a cerebral blood vessel to increase cerebral blood flow, via a change in vessel calibre, in response to a vasoactive stimulus. Measurement of CVR provides information on the functional vascular reserve, an important marker of the health of the cerebrovascular system.

In order to measure CVR, a vasoactive stimulus is administered and the cerebral blood flow response is measured. The most commonly applied vasoactive stimulus is carbon dioxide. When the arterial partial pressure of CO_2_ (PaCO_2_) increases, CO_2_ diffuses across the blood brain barrier^50,51^ and causes hyper-polarisation of smooth muscle and endothelial cells. This results in smooth muscle relaxation and an increase in the release of endothelial factors such as nitric oxide, and subsequent vasodilation. Direct measurement of PaCO_2_ is not possible using non-invasive methods. End-tidal CO_2_ correlates with direct measurement of PaCO_2_ via arterial blood gas sampling^52^ and is therefore used as a surrogate marker of PaCO_2_.^53^ Hypercapnia can be induced by administering a gas mixture containing 5% CO_2_, 21% O_2_ and balanced nitrogen. Recently, computerised gas delivery systems have been developed that can prospectively target end-tidal CO_2_^54,55^ in an effort to standardise and improve repeatability of CVR measurements. However, specialist respiratory circuits are not a prerequisite to CVR measurement. Hypercapnia can be induced as simply as through a breath-hold, with ETCO_2_ measured via nasal cannulae.

The response to vasoactive simulation can be measured using a variety of methods. These include transcranial Doppler ultrasound measurement of the change in blood velocity and vessel diameter, and phase-contrast MRI, but both of these techniques are limited to assessing CVR in individual arterial territories,^56,57^ unlike ASL or BOLD fMRI that can map across the whole brain.⁠ CVR measurements require at least two estimates of CBF. MRI, therefore, offers an advantage over PET, SPECT and CT^58,59^ in that it does not require the use of ionising radiation. In the research setting, the majority of CVR studies are based on BOLD MRI,^59^ because of its widespread availability, high sensitivity, and high spatial and temporal resolution.^60^ However, BOLD is a not a specific measure of cerebral blood flow^61^ ⁠and therefore only provides a semi-quantitative measurement of CVR. BOLD-CVR and ASL-CVR are generally in good agreement with each other,^59^ but ASL has the advantage of providing a quantifiable measure of CBF,^62^ with the caveat that the contrast to noise ratio (CNR) for detecting changes in CBF is typically lower than BOLD.

In order to calculate CVR, the BOLD or ASL signal is measured at the subject’s baseline end-tidal CO_2_ and during hypercapnia. The proportional BOLD or CBF response is then calculated and expressed as either a percentage change in BOLD signal or a percentage change in CBF per mmHg increase in end-tidal CO_2_.

### Applications

CVR correlates with angiographic measures of disease severity both in atherosclerotic and Moyamoya patients^63^ and could therefore serve as an adjunct to angiography in selecting patients for revascularisation therapy. Measurement of CVR could also help in the assessment of the rehabilitation potential of brain regions affected by ischaemic stroke.^64^ CVR could be useful as a marker of neurovascular reserve in a variety of cerebrovascular diseases. For example, CVR measurements correlate with cognitive performance in patients with mild cognitive decline^65,66^ with the severity of regional arterial stenosis in Moyamoya,^67^ and with the severity of motor symptoms in Parkinson’s disease.^68^

The assessment of brainstem CVR could be useful in pre-surgical planning when the neurovascular coupling that underpins the BOLD signal response is compromised by brain pathology. For example, Pillai et al demonstrated that low-grade gliomas can have preserved cerebral blood flow but impaired CVR, thus impeding evaluation of functional activity adjacent to the tumour due to the resultant neurovascular uncoupling^49,69^ This additional knowledge on the state of the local vasculature may help determine the necessity of intra-operative cortical mapping. BOLD-CVR has also been used intraoperatively to determine whether there has been an immediate improvement in cerebral haemodynamics following arterial bypass graft surgery.^70^ The same technique has also been applied to assess CVR following tumour resection, and areas of impaired CVR have been shown to correlate with tumour recurrence on subsequent post-operative contrast-enhanced *T*_1_-weighted scans.^70^

CVR measurements are reproducible using both 1.5T and 3T MRI^71,72^ and are safe and well-tolerated in the clinical population using a CO_2_ stimulus and BOLD MRI acquisition.^73^ Brainstem ASL-CVR and BOLD-CVR measurements are both feasible at 3T.^62^ Sleight et al’s comprehensive review of CVR measurement approaches could serve as a foundation for the application of CVR as a clinical tool.^74^

## Challenges of brainstem functional MRI

The application of the above techniques in the brainstem is challenging for several reasons. Achieving sufficient signal-to-noise (SNR) and thus spatial resolution to image the sub-millimetre internal architecture of the brainstem is time-consuming.^75^ However, the move towards higher MRI field strengths, 3T in the clinical environment and 7T largely as a research tool, helps to address this problem. The brainstem’s caudal location places it away from the elements of typical MRI receiver head coils, further compromising SNR. The susceptibility-induced gradient in the static magnetic field of the scanner caused by air-tissue interfaces in the nearby paranasal sinuses causes distortions and signal drop out.^76^ Gradient-echo echo planar imaging (GE-EPI) is almost ubiquitous for BOLD fMRI and is commonly used for ASL, but it is especially vulnerable to such artefacts.^77^ For ASL in particular, these problems can be partially addressed using alternative readouts such as 3D spiral,^78^ together with parallel imaging, which reduces image acquisition time and susceptibility-induced distortion.^77^

**Figure 5.**
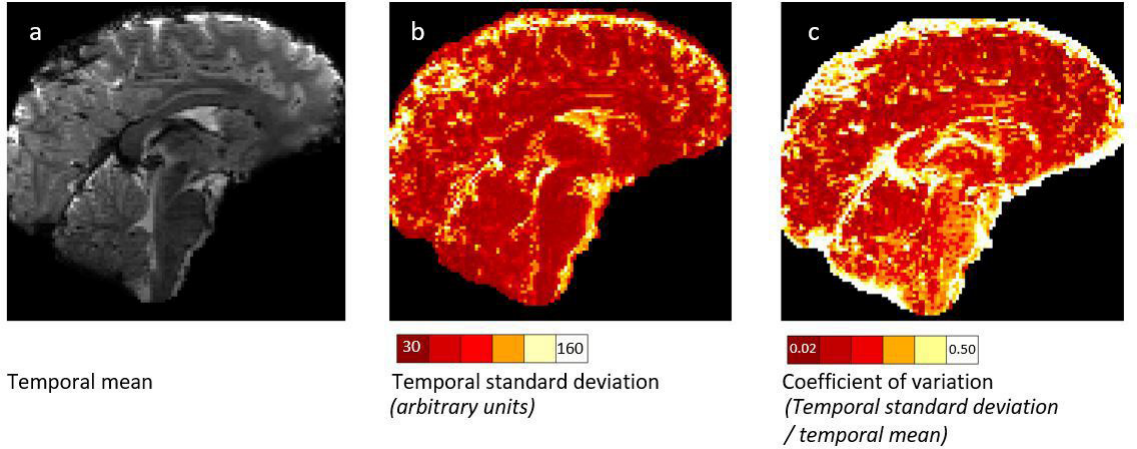
Temporal mean (**a**), temporal standard deviation (**b**) and coefficient of variation (**c**) of a 7T resting state BOLD fMRI acquisition demonstrates the high temporal variance in resting state fMRI signal in the brainstem and surrounding structures.

The brainstem is especially subject to physiological noise (Figure 5). The change in the volume and position of the lungs during respiration causes fluctuations in the static magnetic field present at the brainstem.^79^ This causes misregistration of MRI signal and subsequent anatomic distortion. This effect decreases with the cube of the distance from the lungs, and therefore the brainstem is more strongly affected than the cerebral hemispheres.^75^ Head movement, physiological tissue displacements due to pressure pulsations from the adjacent vertebrobasilar artery or CSF pulsation can cause BOLD and ASL artefacts and misalignment of ASL tag-control volumes.^80,81^ The brainstem is especially vulnerable to physiological pulsations; motion of structures across the cardiac cycle are of the order of 0.5 mm close to the brainstem compared to 0.05 mm in the cerebral cortex.^82,83^

Moving towards higher static magnetic fields can increase SNR which can be traded for an increase in spatial resolution which is necessary to better resolve brainstem nuclei. However, the stronger static magnetic field may introduce problems of its own. These include less uniform transmit and receive radiofrequency fields,^84^ stronger field gradients at air-tissue interfaces exacerbating susceptibility-induced artefacts^84,85^ and increased soft tissue energy deposition^85^ associated with the higher Larmor frequency. Furthermore, physiological noise is proportional to the square of the magnetic field strength.^77^ Physiological noise can be addressed prospectively using navigator methods which track brain motion during the MRI acquisition^86^ or through cardiac gating.^81^ Alternatively, physiological parameters such as heart and respiratory rate can be recorded using physiological monitoring and a retrospective correction applied^87^ or data-driven methods may be applied based on the spatio-temporal characteristics of physiological noise.^77^ Applying a brainstem mask to remove areas of high physiological noise such as the surrounding CSF and vascular spaces can also improve sensitivity to detect functional activity.^88^ Anatomical identification of brainstem nuclei to aid functional localisation is also challenging, although techniques such as magnetisation transfer imaging have been used to identify certain brainstem nuclei.^89^

## Conclusion

Despite the above challenges, functional MRI techniques based on ASL and BOLD can be used to non-invasively investigate neuronal activity and cerebral haemodynamics across the brain. They have been applied to study a variety of disease processes and are already used in certain clinical circumstances. Brainstem functional MRI is challenging, but with further optimisation could feasibly be applied in the clinical setting. Many of the techniques described in this review are feasible in the brainstem using existing 3 Tesla scanners, which are increasingly prevalent in the clinical setting. At 3T, ASL could be applied to map brainstem perfusion in acute ischaemic stroke, as well as estimating brainstem tumour haemodynamics to guide diagnosis and to assess response to treatment. CVR could be useful as a prognostication tool when brainstem haemodynamics are compromised. Finally, higher field strength scanners may render methods such as the mapping of brainstem neural networks using BOLD interpretable at an individual level.
